# Cobalt phosphide-loaded biochar synthesis using phosphate-accumulating yeast and its application as an electrocatalyst

**DOI:** 10.1016/j.btre.2025.e00874

**Published:** 2025-01-09

**Authors:** Yoshihiro Ojima, Riho Akiyoshi, Itto Tokiwa, Takashi Nakazono, Yusuke Yamada, Masayuki Azuma

**Affiliations:** a Department of Chemistry and Bioengineering, Osaka Metropolitan University, 3-3-138, Sugimoto, Sumiyoshi-ku, Osaka 558-8585, Japan; b Research Center for Artificial Photosynthesis, Osaka Metropolitan University, 3-3-138, Sugimoto, Sumiyoshi-ku, Osaka 558-8585, Japan

**Keywords:** *Saccharomyces cerevisiae*, Cobalt phosphate-loaded biochar, Hydrogen evolution reaction, Nitrate reduction reaction

## Abstract

A phosphorus (P)-accumulating mutant strain of *Saccharomyces cerevisiae* is used as a precursor for transition metal phosphides (TMPs) biochar. Tetrahydrofuran treatment of the P-accumulating yeast coupled with pyrolysis resulted in the formation of CoP-loaded biochar (CoP@P-yeast) unlike previously reported Co_2_P-loaded biochar using dry baker's yeast. The CoP@P-yeast exhibited the electrocatalytic activity for the hydrogen evolution with an overpotential of −192 mV at 10 mA cm^−2^. Furthermore, the CoP@P-yeast showed the highest ammonia production rate of 33 mg-NH_3_ h^−1^ mg-catalyst^−1^ in nitrate reduction reaction, as well as much higher than that with platinum on graphitized carbon. Scanning electron microscopy and transmission electron microscopy observations revealed that relatively large TMP crystals mainly located at the biochar surface, which may be beneficial to avoid catalytic deterioration during the nitrate reduction reaction. This study demonstrates that P-accumulating mutant strain of yeast is a suitable precursor to improve the activity of the resulting TMP biochar.

## Highlights

• Tetrahydrofuran treatment of the P-accumulating yeast coupled with pyrolysis resulted in the CoP-loaded biochar (CoP@P-yeast) formation.
• The CoP-@P-yeast exhibited the electrocatalytic activity for the hydrogen evolution with an overpotential of −192 mV at 10 mA cm^−2^.
• The CoP-@P-yeast showed the highest ammonia production rate of 33 mg-NH_4_ h^−1^ mg-catalyst^−1^ in nitrate reduction reaction.

## 1 Introduction

Transition metal phosphides (TMPs) are important hydroprocessing catalysts for desulfurization, denitrogenation, and deoxygenation of oil feedstocks [1,2]. TMPs have also been used as co-catalysts in photocatalysis [3,4], electrocatalysis for water splitting [5,6], and materials for energy storage [7]. Recently, TMPs have been identified to act as nonprecious metal catalysts for the hydrogen evolution reaction (HER) [8,9]. Well-dispersed nanoparticles of TMPs, including Co, Ni, and Fe, exhibit high HER activity with extraordinary mechanical strength and chemical stability. Consequently, they are promising alternatives for noble-metal catalysts. In addition to HER, cobalt phosphide has also been investigated for NO*_x_*^−^ reduction to produce ammonia under strong alkaline condition [10]. The introduction of phosphorus (P) to Co improves the catalyst stability by decreasing the conversion of Co to Co(OH)_2_ during NO_3_^−^ reduction, reducing the free energy change of the reaction for the rate-determining step, and optimizing the energy barrier for the reaction [11].

Traditional methods for TMPs preparation commonly use toxic or hazardous P precursors (e.g., trioctylphosphine, triphenylphosphine, tri-*n*-octylphosphine oxide, red P, and NaH_2_PO_2_) [5,7], which may cause environmental pollution and increase preparation costs. In 2017, Zhang et al. demonstrated the feasibility of using P in microorganisms for metal phosphide formation [12]. Most of the P in microorganisms exists in the stable form of phosphate. They used yeast biomass as an inexpensive and environmentally benign precursor to synthesize TMP biochar as electrocatalysts for HER. It was also demonstrated that the hydrogen evolution on the TMP biochar follows the Volmer-Heyrovsky mechanism [12]. Furthermore, Tong et al. fabricated Co_2_P–C composites using *Candida utilis* cultured in P-rich condition and Co^2+^ as precursors in a simple hydrothermal treatment–pyrolysis. The Co_2_P–C was used for efficient removal of bisphenol A with the peroxymonosulfate because of synergistic pollutant adsorption and oxidation via both radical and nonradical pathways [13]. Although TMPs have been obtained using yeast precursors, an approach involving P-accumulating mutant strain has not been reported for the improvement of TMP-biochar synthesis methods.

The phosphatase (PHO) regulon mutant strain of *Saccharomyces cerevisiae* NOF-1, which constitutively expresses *PHO81*, reportedly accumulates P in its cells under high-phosphate conditions [14]. In a previous quantitative analysis, we found that the contribution of P to the total dry weight of the cell reached approximately 8.5 %, which was much higher than that of parent strains of yeast (1-–2 %) [15] and P-rich *C. utilis* (4.3 %) [13]. We also found that 60 % of the total P in the cells accumulated as polyphosphate (Poly-P). It is also unique characteristic that > 90 % of poly-P is localized to vacuoles in P-accumulating *S. cerevisiae* [16].

This study aimed to investigate the effect of P-accumulation in yeast mutant cells on the resulting catalyst and evaluate the performance of the obtained cobalt phosphide as an electrocatalyst. We selected P-accumulating *S. cerevisiae* as a precursor for cobalt phosphide/carbon composite formation by a combined tetrahydrofuran (THF) treatment and pyrolysis approach. The catalytic activities of the obtained materials were evaluated in the HER and nitrate reduction reaction (NO_3_^−^RR) and discussed.

## 2 Materials and methods

### 2.1 Microbial strains, media, and culture conditions

Table 1 summarizes the materials used in this study. Commercial dry baker's yeast (Nisshin Seifun Group, Tokyo, Japan) was used as the control strain. NBW7 is a parental strain of NOF-1. NOF-1 is P-accumulating strain with a point mutation that gives rise to the phenotype *PHO81^c^* [17,18]. Yeast extract–peptone–dextrose (YPD) medium (1 % [w/v] yeast extract, 2 % [w/v] HIPOLYPEPTON, and 2 % [w/v] glucose) was used to culture the yeast strains. KH_2_PO_4_ (0.4 % [w/v]) was added to the YPD medium to generate phosphate-rich YPDP medium. Cells were precultured overnight at 30 °C in flasks containing 100 mL of YPDP medium and then inoculated into a jar fermenter VF-3z (TIYODA Manufacturing Co., Ohta, Japan) containing 2 L of YPDP medium. Aeration in the fermenter was controlled at 1 vol of air per volume of medium per minute (vvm) at 30 °C with agitation at 250 rpm. After 24 h, the cells were collected by centrifugation (3000 × g, 5 min), washed twice with pure water, and freeze-dried for 2 days.

**Table 1:** Materials used in this study.

Precursor for biochar	P content (%)	Function	Reference
Yeast	0.9	Commercial dry baker's yeast	Ojima et al. 2019 [19]
Phosphorylated yeast	3.4	Chemically phosphorylated dry baker's yeast	Ojima et al. 2019 [19]
Parent strain of P-yeast	2.0	*S. cerevisiae* NBW7 (MATa ade2 his3 leu2 trp1 ura3 pho3-1)	Ojima et al. 2023 [15]
P-accumulating yeast (P-yeast)	8.5	*S. cerevisiae* NOF-1 (MATa ade2 his3 leu2 trp1 ura3 pho3-1 PHO81^c-^1)	Ojima et al. 2023 [15]
Nucleic acid (NA)	6.1	NA extracted from fresh yeast cells	Tong et al. 2020 [20]

### 2.2 Phosphorylation of dry baker's yeast cells

Commercial dry baker's yeast was phosphorylated (phosphorylated yeast) following the methods reported in a previous study [19]. Briefly, yeast cells were washed five times with pure water and then fixed with 70 % (v/v) ethanol for 2 h The yeast cells were phosphorylated using a 20 % sodium cyclotriphosphate hexahydrate solution at 50 °C for 20 h The pH of the solution was maintained at 12 by adding 3 M aqueous sodium hydroxide with stirring. After the reaction reached completion, the phosphorylated yeast cells were washed with distilled water and lyophilized in a freeze-drier FDU-1200 (EYELA, Tokyo, Japan)

### 2.3 Nucleic acid extraction from fresh yeast

Nucleic acid (NA) extraction from yeast (*S. cerevisiae*) was conducted according to a previous report [20]. Briefly, the NA extraction from fresh yeast (TOMIZ CUOCA, Tokyo, Japan) was performed using a concentrated salt solution. Typically, 100 g of dry yeast powder was suspended in 0.9 L of 72.2 g L^−1^ NaCl with constant stirring. The solution pH was adjusted to 7.5 using NaOH or HCl. The suspension was stirred continuously in a water bath (85 °C) for 4 h, and then rapidly cooled to below 10 °C in an ice bath. Cell residues were removed by centrifugation at 4000 rpm for 10 min. The pH of the supernatant was adjusted to 2.5 to allow for precipitation of nucleic acids. After centrifugation and washing with ethanol three times, the precipitated nucleic acids were lyophilized. The resulting product is referred to as extracted NA.

### 2.4 Preparation of the catalysts and X-ray diffraction analyses

First, 0.3 g of cobalt nitrate hexahydrate and 0.25 g of freeze-dried yeast cells or extracted NA were dissolved in 2.5 mL of deionized water. Tetrahydrofuran (THF, 5.5 mL) was added to obtain a cosolvent environment with a THF/water volume ratio of 5:11 [21]. After stirring for 2 h at room temperature, a purplish-brown solid product was isolated by centrifugation (3000 × g, 5 min) and then freeze-dried for 24 h The resultant product was subjected to pyrolysis (carbonization) at 900 °C for 0.5–2.0 h in a tubular reactor under a flow of nitrogen gas (15 or 500 mL min^−1^). After leaving the reactor to cool to room temperature, a black carbonized product was obtained.

X-ray diffraction (XRD) data for the carbonized products were collected at 23.0 °C with an X-ray diffractometer RAXIS-RAPID Imaging Plate (Rigaku, Tokyo, Japan) using Cu Kα radiation (*λ* = 1.54178 Å).

### 2.5 Hydrogen evolution reaction

All the electrochemical measurements were performed using a three-electrode system (Automatic Polarization System HSV-110, Hokuto Denko Co., Tokyo, Japan) in 0.5 M H_2_SO_4_ electrolyte. Platinum on graphitized carbon (20 % mass fraction of Pt, Sigma–Aldrich, USA) was used as the control. Typically, 3 mg of catalyst and 30 μL of 5 % Nafion solution (Sigma–Aldrich) were dispersed in 1.2 mL of ethanol solution by sonicating for 1 h to form a homogeneous ink. Next, 30 μL of the dispersion (containing 75 μg of catalyst) was loaded onto a glassy carbon electrode (ø 5 mm, loading: 0.37 mg cm^−2^). The reference and counter electrodes were Ag/AgCl (in 3 M NaCl solution) and Pt, respectively. Linear sweep voltammetry was performed in 15 mL of 0.5 M H_2_SO_4_ with the scan rate of 5 mV s^−1^. All potentials were calibrated to the reversible hydrogen electrode (RHE). The working electrode was polished using Al_2_O_3_ powders with sizes down to 0.05 μm. The rotating disk electrode (RDE) measurements were carried out using a rotating electrode system (Dynamic Electrode HR-201 and Dynamic Electrode Controller HR-202, Hokuto Denko Co.) linked to a potentiostat (EC stat 302, EC Frontier Co. Ltd, Kyoto, Japan). A three-electrode cell was employed with the RDE consisting of a GC disk electrode and platinum coil as a counter electrode and SCE as a reference electrode. The controlled-potential electrolysis at –0.45 V vs. SCE was performed in an Ar-saturated 0.5 M H_2_SO_4_ with rotating rates of 1000 rpm.

### 2.6 Nitrate reduction reaction

The electrochemical measurements were performed using a standard three-electrode system (Automatic Polarization System HSV-110, Hokuto Denko Co.) in an H-type cell separated by a Nafion 117 proton exchange membrane (DuPont). The cathode and anode chambers of the H-type cell contained 50 mL of aqueous solutions (1 M NaOH and 1 M NaNO_3_). A glassy carbon electrode loading a sample catalyst was prepared by the same procedure as in the HER (loading: 0.22 mg cm^−2^). This was used as the working electrode in the cathode chamber. A graphite rod was used as the counter electrode in the anode chamber, and the saturated calomel electrode was used as the reference electrode in the cathode chamber. Chronoamperometry was conducted at -0.25 V in the electrolyte for 3 h. All potentials measured against the Hg/HgO electrode were converted to the RHE scale as follows: *E* (vs. RHE) = *E* (vs. saturated calomel electrode) + 0.0591 × pH + 0.118 V.

The ammonia concentration in the supernatant was measured using the modified indophenol blue method [22]. Briefly, the indicated reagents were added to the supernatant, which had been appropriately diluted with pure water, and the solution was mixed. After incubating the solution at room temperature for 45 min, the absorbance of the solution was measured at 630 nm using a microplate reader. The ammonia concentrations of the samples were calculated using a standard curve.

### 2.7 X-ray fluorescence spectroscopy and Raman spectroscopy

The atomic ratios of the carbonized products were determined by X-ray fluorescence (XRF) measurements using a PANalytical Epsilon 1 (Malvern Instruments Ltd, UK). Raman spectra were taken on an NRS-4500 Raman spectrometer (JASCO Co, Tokyo, Japan) with a 532 nm excitation laser.

### 2.8 Transmission electron microscopy and scanning electron microscopy with energy-dispersive X-ray spectroscopy

For transmission electron microscopy (TEM), the sample was suspended in 1 mL of ethanol and dispersed for 1 h by ultrasonication (BRANSON1510, Yamato, Tokyo, Japan). The sample solution (10 µL) was dropped onto a mesh copper grid and dried under reduced pressure. The resultant sample was observed by TEM (JEM 2100, JEOL, Tokyo, Japan). The composition of the sample was analyzed using an energy-dispersive X-ray spectrometer (EDS) attached to the TEM instrument. Analyses were conducted using an accelerating voltage of 200 kV, with a dead time of 1–2 % and a collection time of 700 s.

For scanning electron microscopy (SEM), the sample was observed by field emission (FE)-SEM (JSM 6500-FS, JEOL). The composition of the sample was analyzed using an EDS attached to the FE-SEM instrument. Analyses were conducted using an accelerating voltage of 25 kV, with a dead time of 22–30 % and a collection time of 60 s.

### 2.9 Specific surface area and conductivity

Nitrogen adsorption measurements were performed at −196 °C on a Belsorp-Mini X (MicrotracBEL, Osaka, Japan). The specific surface area (SSA) was determined from the linear part of the Brunauer–Emmett–Teller plot.

The sample conductivity was measured in a dry closed system. The sample was pressed into a pellet (ø 5 mm, thickness: 1 mm). The voltage and current were measured using a potentiostat (BioLogic SP-300, TOYO Corp., Tokyo, Japan) and the resistance was calculated.

### 2.10 Statistical analysis

Each result is presented as the mean ± the standard deviation for more than three independent experiments.

## 3 Results and discussion

### 3.1 Characterization of materials

**Fig. 1:**
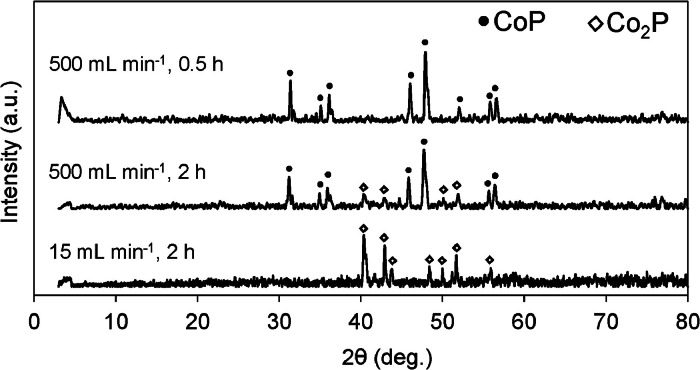
XRD spectra of the catalysts prepared using P-accumulating yeast cells. Catalysts were prepared by pyrolysis at 900 °C in a tubular reactor for various times (0.5 or 2.0 h) under nitrogen (15 or 500 mL min^−1^).

The P-accumulating yeast cells cultured under high-phosphate conditions were freeze-dried and treated in a THF/water cosolvent environment before high-temperature pyrolysis. It has been reported that the THF/water cosolvents efficiently broke down or dissociated the cell wall of plant biomass [21,23]. This treatment is thought to loosen the yeast cell wall, helping the cobalt ions penetrate into the cells. XRD was used to identify the crystal structures of the samples. The Co_2_P sample prepared under N_2_ flow (15 mL min^−1^, 2 h) at a high temperature had an orthorhombic structure (JCPDS card no. 32-0306) (Fig. 1). This structure was consistent with that for the sample using dry baker's yeast prepared under hydrothermal conditions followed by high temperature pyrolysis [12]. This structural similarity suggests that THF/water treatment could be an alternative to hydrothermal treatment without large energy consumption. Meanwhile, N_2_ flow rate was increased to 500 mL min^−1^ during 2 h high-temperature treatment, peaks for Co_2_P and CoP (JCPDS card no. 29-0497) were obtained. Furthermore, the shorter high-temperature treatment time of 0.5 h provided CoP. This is for the first time to successfully prepare CoP using yeast cell as the P-source, unlike the Co_2_P crystal phase reported in previous studies [12,13]. Although the formation mechanisms of the two types of cobalt phosphide are not fully understood, a high P content (8.5 %) in the P-accumulating yeast, and high-temperature treatment in short time (0.5 h) with high N_2_ flow rate (500 mL/min) appears to be important for the CoP synthesis.

**Fig. 2:**
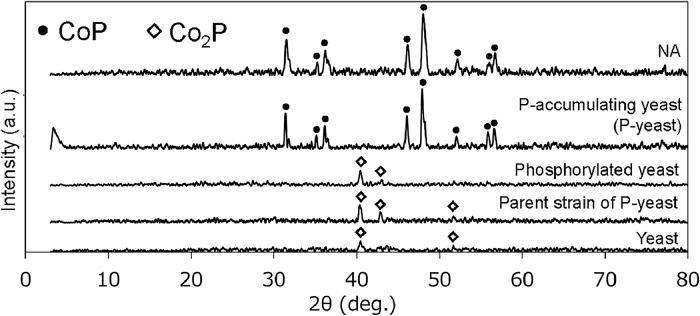
XRD spectra of the catalysts prepared using the various types of yeast cells. Catalysts were prepared by pyrolysis at 900 °C for 0.5 h in a tubular reactor under nitrogen (500 mL min^−1^).

To confirm the effect of the biomass P content on the formation of CoP, different types of yeast cells were treated under the same conditions (THF/water, 500 mL min^−1^ N_2_, 900 °C for 0.5 h). The formation of Co_2_P was confirmed with three strains (Fig. 2): the parent strain of P-accumulating yeast NBW7 (2.0 % P), commercial dry baker's yeast (0.9 % P), and chemically phosphorylated dry baker's yeast (3.4 % P) [19]. In an earlier report, CoP was synthesized using nucleic acid (NA) extracted from yeast cells as the P source [20]. In the present study, NA was extracted from fresh yeast and treated under the same conditions. A peak for pure CoP was observed as previously reported (Fig. 2). The P content of NA extracted from yeast cells is reportedly 6.1 % [20]. Considering that P-rich *C. utilis* (4.3 %) produced Co_2_P in a previous study [13], a P content of 5 % or more is considered important for CoP formation.

In addition to differences in P content, the yeast strains and culture conditions were also different in the above experiments. Therefore, we investigated whether addition of extracellular phosphate to dry baker's yeast during THF/water treatment contributed to CoP formation. With addition of < 0.4 M phosphate, the formation of Co_2_P was evidenced by XRD (Fig. S1). When the concentration of phosphate was increased to 0.6 M, diffraction peaks assignable to Co_2_P and CoP were observed, which suggested that the addition of extracellular phosphate also induced the CoP formation. When the concentration of extracellular phosphate was increased further to 1.6 M, Co_2_P/CoP was also obtained. In the case of NA, it was proposed that phosphate groups in the backbone and amino groups in the bases of nucleic acids could possibly coordinate with cobalt ions to form a Co–nucleic acid complex precipitate [20]. Most of the phosphate in P-accumulating yeast exists as poly-P [15], which suggests that the presence of phosphate in the form of polymers, such as poly-P or NA, is important for the synthesis of CoP via Co–phosphate complex formation.

### 3.2 Electrocatalytic hydrogen evolution reaction

**Fig. 3:**
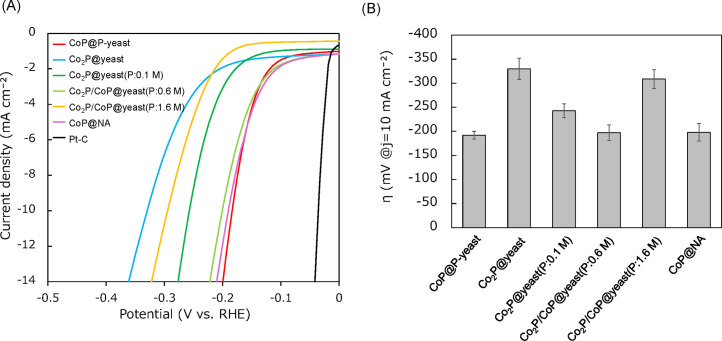
Evaluation of the activity as an electrode catalyst in the HER (tested in 0.5 M H_2_SO_4_ at a scan rate of 5 mV s^−1^). (A) Polarization curves of the prepared catalysts. (B) The overpotential of each sample at a cathodic current density of 10 mA cm^−2^.

Catalysts developed from P-accumulating yeast (CoP@P-yeast), NA (CoP@NA), dry baker's yeast (Co_2_P@yeast), and dry baker's yeast with extracellular phosphate (Co_2_P/CoP@yeast(P:0.1 M, 0.6 M, and 1.6 M)) were further examined as the electrocatalysts for HER. Fig. 3A shows the HER polarization curves obtained with the different samples in 0.5 M H_2_SO_4_. 20 % Pt-C was used as a positive control as well as that with three of the catalyst samples (CoP@P-yeast, CoP@NA, and Co_2_P/CoP@yeast(P:0.6 M). Successful HER with Pt-C was evidenced by a large increase in the current density at low voltage. The large current densities at the same voltage with three of the catalysts suggested that CoP was present in the carbon matrix and boosted the catalytic activity. The overpotentials (achieved at 10 mA cm^−2^) are compared in Fig. 3B. The overpotential of CoP@P-yeast of -192 mV was much lower than that of Co_2_P@yeast (-330 mV). For dry baker's yeast, the overpotential of -197 mV was recorded for Co_2_P/CoP@yeast(P:0.6 M). The overpotential for Co_2_P/CoP@yeast(P:1.6 M) was -309 mV. CoP@NA exhibited a low overpotential (-198 mV), which was comparable to that of CoP@P-yeast. Higher HER activity of CoP than Co_2_P has been reported previously [24], however, the activity of the CoP@P-yeast proposed in this study is on the same level as that of CoP@NA and Co_2_P/CoP@yeast(P:0.6 M). Therefore, XRF analysis was conducted for Co_2_P@yeast, CoP@P-yeast, Co_2_P/CoP@yeast(P:0.6 M) and CoP@NA. XRF measurements indicated that the difference in weight percent of Co contained in these catalysts were within 10 %, suggesting that Co amount in each catalysts are virtually the same. This is reasonable because the same amount of Co was added during the synthesis of these catalysts. The molar ratio of Co to P in Co_2_P@yeast was found to be 2.21, which is virtually the same as that predicted value 2, within an experimental error. The molar ratios of Co to P in CoP@P-yeast and CoP@NA were 0.94 and 1.06, respectively, supporting that CoP was formed in these catalysts. The molar ratio of Co_2_P/CoP@yeast(P:0.6 M) was 1.06. Although diffraction peaks assignable to Co_2_P and CoP were observed by XRD, XRF measurement revealed that CoP was mainly formed in Co_2_P/CoP@yeast(P:0.6 M). This is the reason why the activities of three catalysts in HER were comparable.

The stability of CoP@P-yeast, Co_2_P/CoP@yeast(P:0.6 M), and CoP@NA showing high activity was examined by the RDE measurements. The voltammograms were measured at –0.45 V in 0.5 M H_2_SO_4_ with rotating speed of 1000 rpm where the influence of formed bubbles covering the electrode surface can be suppressed. Figure S2 shows the relative current value normalized to initial current. In the cases of CoP@NA and Co_2_P/CoP@yeast(P:0.6 M), the currents gradually decreased to 88 and 67 % of the initial currents, respectively, after 1700 s. In contrast, the current obtained with CoP@P-yeast exceeded 95 % of the initial current after 1700 s. Although the overpotentials determined by the LSV measurements of these three catalysts are comparable, CoP@P-yeast exhibited the most superior stability. Furthermore, formation of hydrogen bubble was observed on the electrode modified with CoP@P-yeast. The fluctuation in the current resulted from the generation of hydrogen bubbles.

### 3.3 Electrocatalytic nitrate reduction reaction

**Fig. 4:**
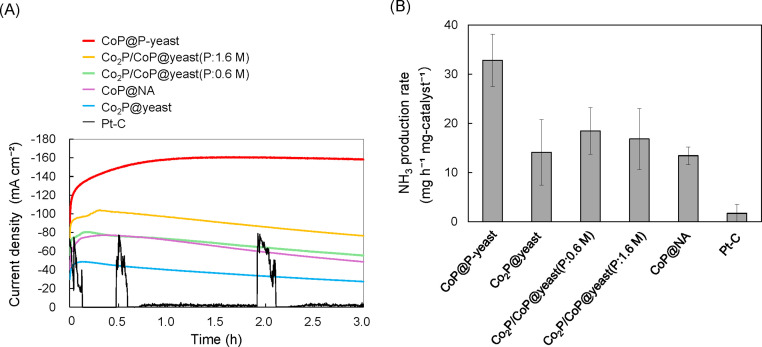
Evaluation of activity as an electrode catalyst in the NO_3_^−^RR (tested in 1.0 M NaOH/1.0 M NaNO_3_ at −0.25 V). (A) Chronoamperometric curves of the prepared catalysts. (B) NH_3_ production rate of each sample.

The catalysts were further evaluated in the NO_3_^−^RR. Fig. 4A shows the chronoamperometric curves of different samples at −0.25 V in 1.0 M NaOH/1.0 M NaNO_3_. Time profile of the current density with the control (Pt-C) was unstable and very low. For Co_2_P@yeast, the catalytic current started at 30 mA cm^−2^ and peaked at 45 mA cm^−2^ after 10 min. These results suggest that CoP-C prepared with yeast cells act as electrocatalysts for the NO_3_^−^RR. This is the first demonstration of the use of a biomass-derived CoP catalyst for NH_3_ production in the NO_3_^−^RR. The catalytic current decreased in a linear manner to 30 mA cm^−2^ at the end of the reaction (3 h). The maximum current density with Co_2_P/CoP@yeast(P:0.6 M), Co_2_P/CoP@yeast(P:1.6 M), and CoP@NA, were higher than those of Co_2_P@yeast, however, the catalytic current decreased linearly over time. The continuous decrease in the catalytic current was attributed to degradation of the catalyst by both the alkaline conditions and NO_3_^−^ in the NO_3_^−^RR [10]. Among the examined catalysts, CoP@P-yeast had the highest starting current (approximately 150 mA cm^−2^), and the current was maintained at the highest value throughout the operation. The activity of the CoP@P-yeast in NO_3_^−^RR was higher than that of any of the other catalysts containing CoP.

The production of NH_3_ was also quantified by colorimetric methods (Fig. 4B). The NH_3_ production rate of CoP@P-yeast was 33 mg-NH_3_ h^−1^ mg-catalyst^−1^, while these of the other catalysts were between 10 and 20 mg-NH_3_ h^−1^ mg-catalyst^−1^. Table 2 summarizes the NH_3_ production rates obtained using the electrocatalysts in the NO_3_^−^RR. The NH_3_ production rate of CoP@P-yeast in this study was comparable to that of catalysts prepared using other metals or materials [[25], [26], [27], [28]], and higher than that of cobalt phosphide nanosheet arrays grown on carbon fiber cloth based on the evaluation per catalyst weight [10]. In contrast, the Faraday efficiency (FE) with CoP@P-yeast was 67.6 %, which was low compared with those for the other catalysts (close to 100 %). One possible reason for low FE is the by-production of hydrogen from competitive reaction [10].

**Table 2:** Comparison of the NO_3_^−^RR.

	Potential (vs RHE)	FE [%]	Ammonia-evolution rate[μg h^-1^ mg-catalyst^-1^]	References
CoP@P-yeast	-0.25	67.6	32,812	This study
CoP NAs/CFC	-0.3	100	9690	Ye, *et al*., (2022) [10]
Cu nanosheet	-0.15	99.7	390	Fu *et al*., (2020) [25]
Strained Ru nanoclusters	-0.2	100	94,686	Li, *et al*., (2020) [26]
CoOx	-0.3	93.4	82,400	Wang, *et al*., (2021) [27]
Fe SAC	-0.85	67	20,000	Wu, *et al*., (2021) [28]

### 3.4 Characterization of the constructed catalysts to determine the cause of high catalytic activity of CoP@P-yeast in the NO_3_^−^RR

The degree of graphitization of CoP@P-yeast was evaluated by Raman spectrum (Fig. S3). Raman spectra of CoP@P-yeast and the other materials showed two peaks: the first one in the range of 1320 to 1360 cm^-1^, called as d-band, and the second peak ranging from 1500 to 1600 cm^-1^, called as G-band [29,30]. Because the two peaks are separated but broad, indicating a relatively low graphitic degree of the formed carbon, which is consistent with first report of TMP biochar [12]. The catalyst conductivity was further evaluated because conductivity is important for an electrocatalyst. The conductivity results are summarized in Table S1. The conductivity of the commercial Pt-C was 0.174 S cm^−1^, which was similar to the that of carbon black [31]. This result indicates that the conductivity measurements are accurate in the present study. The conductivity of CoP@P-yeast was 0.085 S cm^−1^, which was higher than that of Co_2_P@yeast (0.046 S cm^−1^) but lower than that of Pt-C. The Co_2_P/CoP@yeast(P:0.6 M) had the highest conductivity (0.244 S cm^−1^), and CoP@NA had the lowest (0.003 S cm^−1^). Thus, there was no clear correlation between the conductivity and the electrocatalytic activity in the HER and NO_3_^−^RR.

The BET surface area (SA) of CoP@P-yeast was 278 m^2^ g^−1^, which was higher than that of Pt-C (125 m^2^ g^−1^) and comparable to that of CoP@NA (249 m^2^ g^−1^). Although there were differences among the BET SAs, no correlation was observed with catalytic activity.

**Fig. 5:**
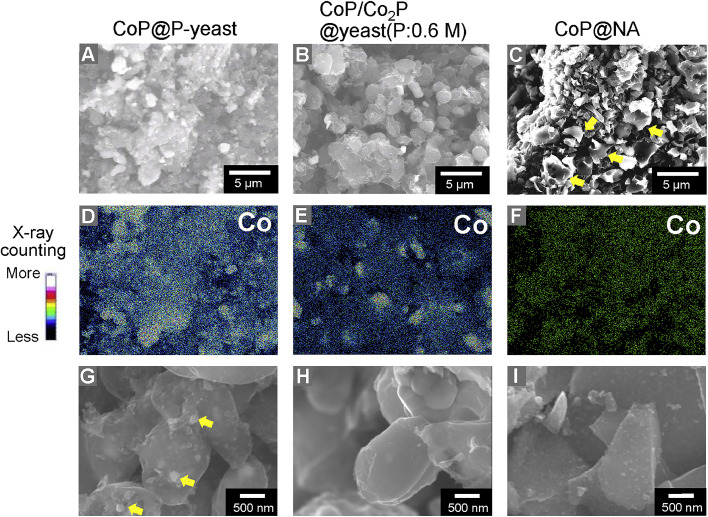
SEM images and two-dimensional localization of cobalt on the prepared catalysts. (A-C) SEM image of the prepared catalysts. The arrows indicated the flake structure. (D-F) Two-dimensional localization of cobalt from FE-SEM/EDS. (G-H) Magnified SEM images of the prepared catalysts. The arrows indicated the large CoP crystals.

To further evaluate the cause of the high catalytic activity of CoP@P-yeast in the NO_3_^−^RR, SEM observation was conducted for CoP@P-yeast, Co_2_P/CoP@yeast(P:0.6 M) and CoP@NA. All samples were placed in the vacuum chamber of a standard FE-SEM equipped with EDS. The SEM images of the samples are shown in Fig. 5. Both CoP@P-yeast and Co_2_P/CoP@yeast(P:0.6 M) were spherical particles with diameters of approximately 2ｰ3 µm, which corresponded to the size of a dehydrated yeast cell (Fig. 5A, B). Clusters formed by aggregation of multiple cells were also observed in these samples. By contrast, CoP@NA showed the flake structure as indicated by arrows (Fig. 5C). These flakes were obtained because the extracted NA did not have a specific shape like yeast cells. Cobalt peak was detected in EDS spectra from the region containing the sample. Typical result for the two-dimensional localization of Co element is shown in (Fig. 5D-F). The density of cobalt was higher in CoP@P-yeast than in Co_2_P/CoP@yeast(P:0.6 M) (Fig. 5D and E). Furthermore, the density of cobalt in CoP@NA was much lower than in the other samples (Fig. 5F). The peak intensities in the XRD patterns were all approximately the same (Fig. 2 and Fig. S1), which suggested that CoP crystals were localized on the surface of the CoP@P-yeast. Magnified image showed that large CoP crystals existed on CoP@P-yeast sample (Fig. 5G), whereas those are not seen in the other two samples (Fig. 5H and I). These results suggest that large CoP crystals localize on the surface of CoP@P-yeast.

**Fig. 6:**
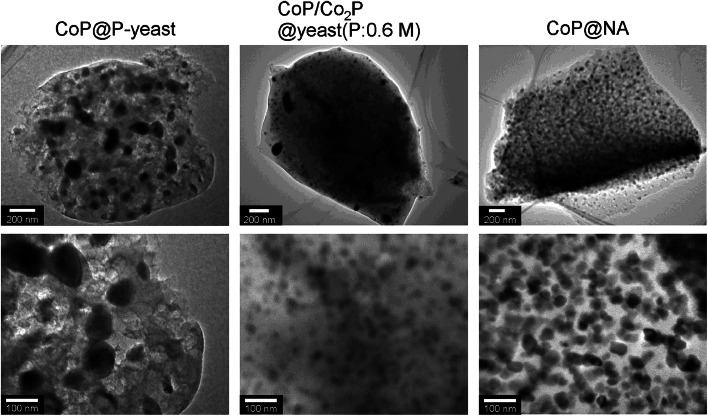
TEM images of the prepared catalysts.

TEM observation was conducted for CoP@P-yeast, Co_2_P/CoP@yeast(P:0.6 M), and CoP@NA. Fig. 6 shows a dark-field TEM image of a randomly selected sample. In the case of CoP@NA, small CoP crystals were highly dispersed in this carbon matrix (Fig. 6). For catalysts using yeast cells as a support, larger crystals were embedded inside the carbon matrix. The diameter of the largest crystal was > 100 nm in Co_2_P/CoP@yeast(P:0.6 M). In the case of CoP@P-yeast, the CoP crystals were even larger (Fig. 6), and some were > 500 nm in diameter (Fig. S4A-C). TEM-EDS revealed that these large crystals were also cobalt phosphide (Fig. S4D-F).

While the NO_3_^−^RR using CoP was performed under alkaline conditions to suppress the competitive HER, CoP surface was oxidized to Co(OH_2_) and the catalytic activity decreased [10]. CoP@P-yeast contained larger CoP crystals than the other catalysts and these crystals were localized on the surface of the carbon matrix. It is thought that this crystalline state of CoP on biochar stabilized the catalytic activity and maintained the catalytic current during the NO_3_^−^RR. Thus, CoP@P-yeast is an excellent NO_3_^−^RR catalyst that is inexpensive and easy to prepare.

## 4 Conclusions

In this study, P-accumulating *S. cerevisiae* mutant strain was used as precursor to prepare a catalyst. THF treatment coupled with pyrolysis produced cobalt phosphide/carbon composites (CoP@P-yeast). The developed CoP@P-yeast performed well as a catalyst for HER with an overpotential of −192 mV at 10 mA cm^−2^, which was almost equal to that of CoP@NA and much lower than that of Pt-C. CoP@P-yeast had the highest ammonia production rate among the biochar composites (33 mg-NH_3_ h^−1^ mg-catalyst^−1^), which was much higher than that of Pt-C. Catalyst deterioration during the reaction, as seen with other catalysts, was not observed for CoP@P-yeast. The stability was thought to be caused by localization of relatively large TMP crystals on the catalyst surface in CoP@P-yeast. This study shows that P-accumulation in *S. cerevisiae* mutant cells improves the activity of the resulting biochar catalyst.

## Ethics approval

All experimental procedures did not include any animal or human element.

## CRediT authorship contribution statement

**Yoshihiro Ojima:** Writing – original draft, Validation, Supervision, Resources, Project administration, Methodology, Funding acquisition, Formal analysis, Data curation, Conceptualization. **Riho Akiyoshi:** Visualization, Validation, Methodology, Formal analysis, Data curation. **Itto Tokiwa:** Methodology, Formal analysis, Data curation. **Takashi Nakazono:** Data curation, Formal analysis, Methodology, Software, Validation, Writing – review & editing. **Yusuke Yamada:** Writing – review & editing, Validation, Methodology. **Masayuki Azuma:** Writing – review & editing, Validation, Supervision.

## Declaration of competing interest

The authors declare no competing interests.

## Data Availability

Data will be made available on request.

## Appendix Supplementary materials

Fig. S1 XRD spectra of the catalysts prepared using dry baker's yeast cells with extracellular phosphate addition. Catalysts were prepared by pyrolysis at 900 °C for 0.5 h in a tubular reactor under nitrogen (500 mL min^−1^). Image, application 1

Fig. S2 The relative current values obtained by RDE measurement. The current was normalized to initial current. RDE measurements were carried out using a rotating electrode system. The controlled-potential electrolysis at -0.45 V vs. SCE was performed in an Ar-saturated 0.5 M H_2_SO_4_ with rotating rates of 1000 rpm. Image, application 2

Fig. S3 Raman spectra of the prepared catalysts. Image, application 3

Fig. S4 TEM images (A, B, C) and two-dimensional localization of cobalt and phosphorus on the prepared catalyst (CoP@P-yeast) analyzed using TEM-EDS (D, E, F). Image, application 4

Image, application 5
