# Supplementary figures and images for: Cobalt phosphide-loaded biochar synthesis using phosphate-accumulating yeast and its application as an electrocatalyst
Source: Biotechnol Rep (Amst). 2025 Jan 9;45:e00874. doi: 10.1016/j.btre.2025.e00874 (PMC11787416; doi:10.1016/j.btre.2025.e00874)

Fig. S1

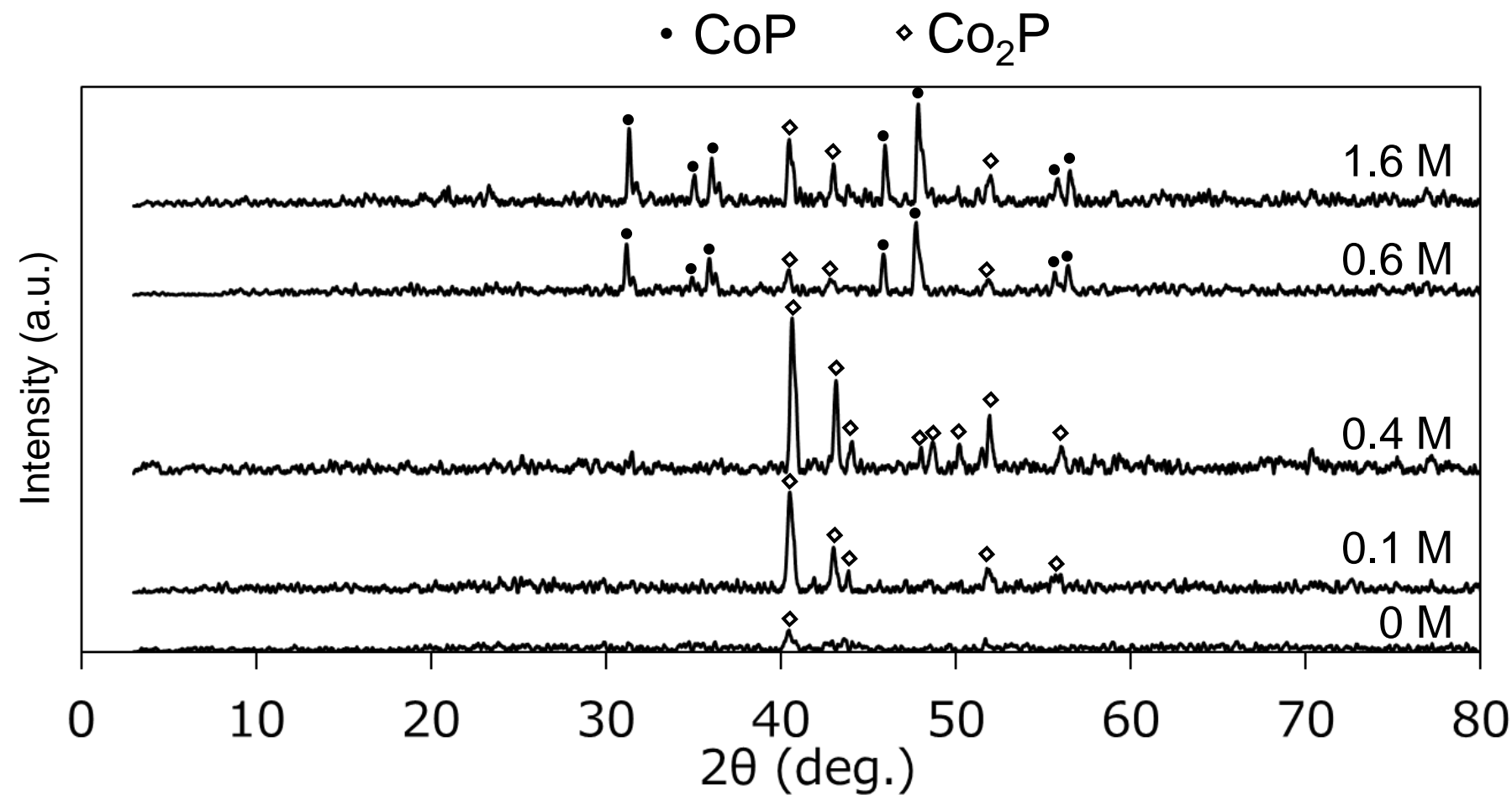

Supplement: Supplementary file 1 — Fig. S1 XRD spectra of the catalysts prepared using dry baker's yeast cells with extracellular phosphate addition. Catalysts were prepared by pyrolysis at 900 °C for 0.5 h in a tubular reactor under nitrogen (500 mL min−1). [file mmc1.pdf]

Fig. S2

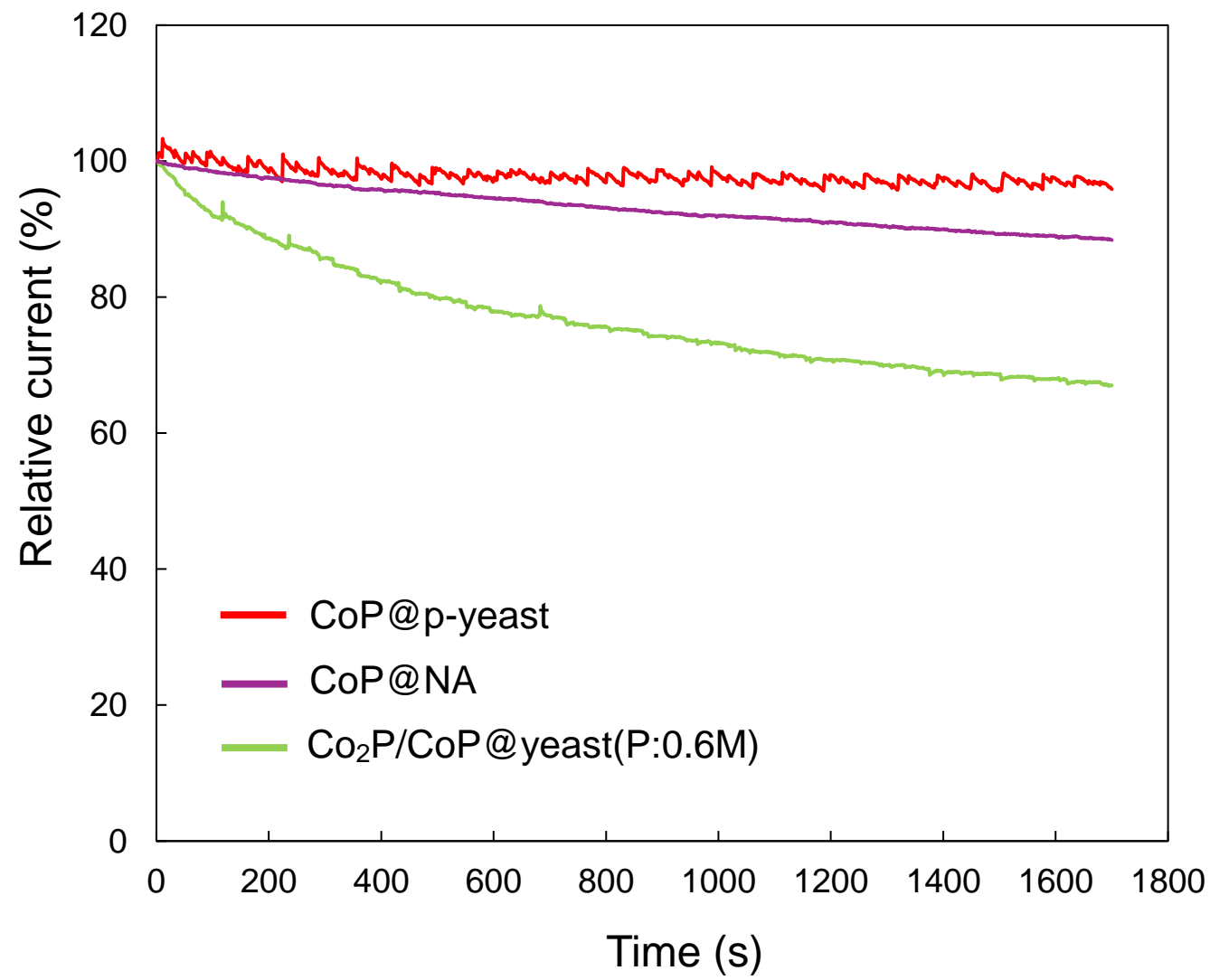

Supplement: Supplementary file 2 — Fig. S2 The relative current values obtained by RDE measurement. The current was normalized to initial current. RDE measurements were carried out using a rotating electrode system. The controlled-potential electrolysis at -0.45 V vs. SCE was performed in an Ar-saturated 0.5 M H2SO4 with rotating rates of 1000 rpm. [file mmc2.pdf]

Fig. S3

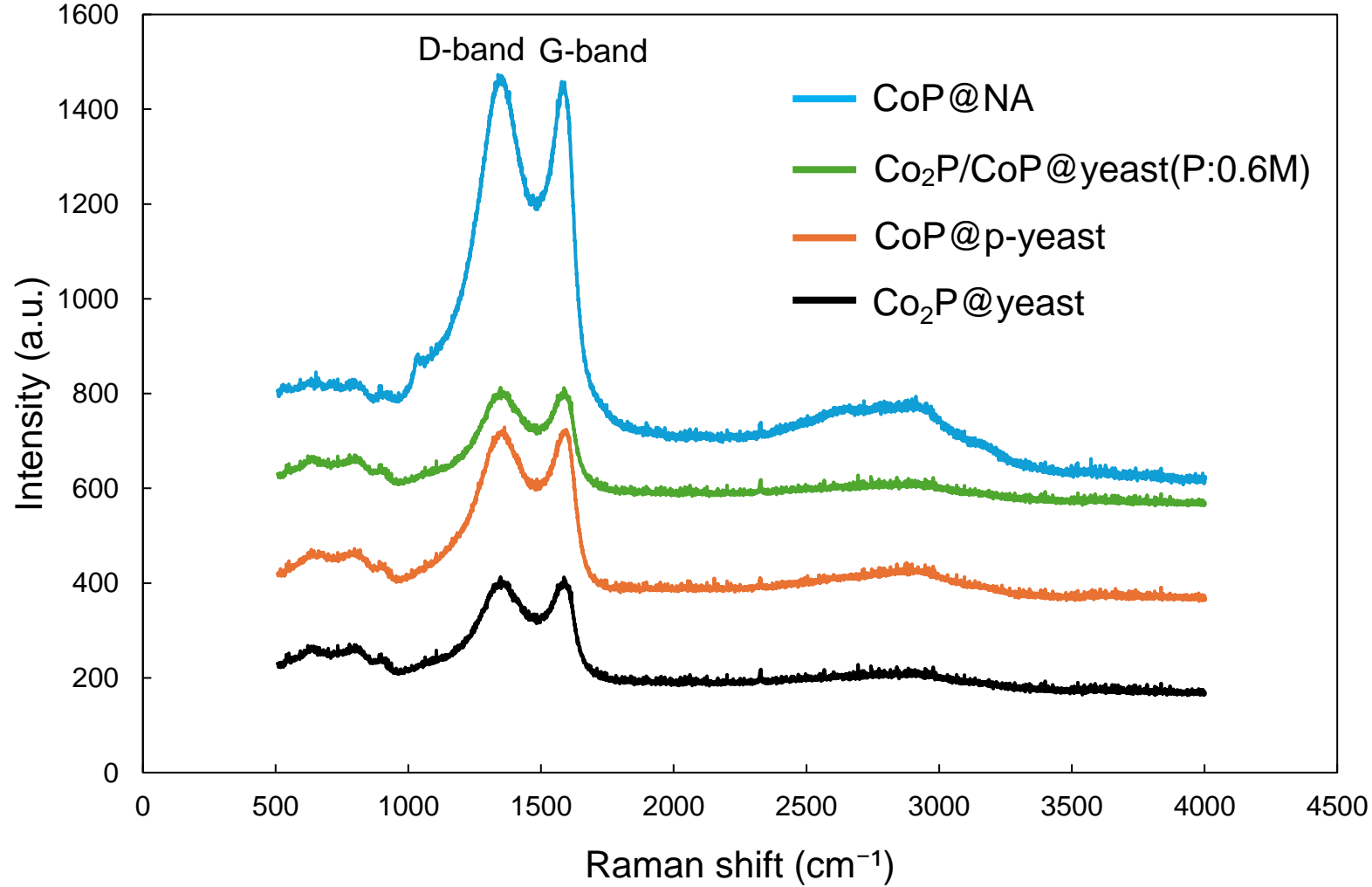

Supplement: Supplementary file 3 — Fig. S3 Raman spectra of the prepared catalysts. [file mmc3.pdf]

Fig. S4

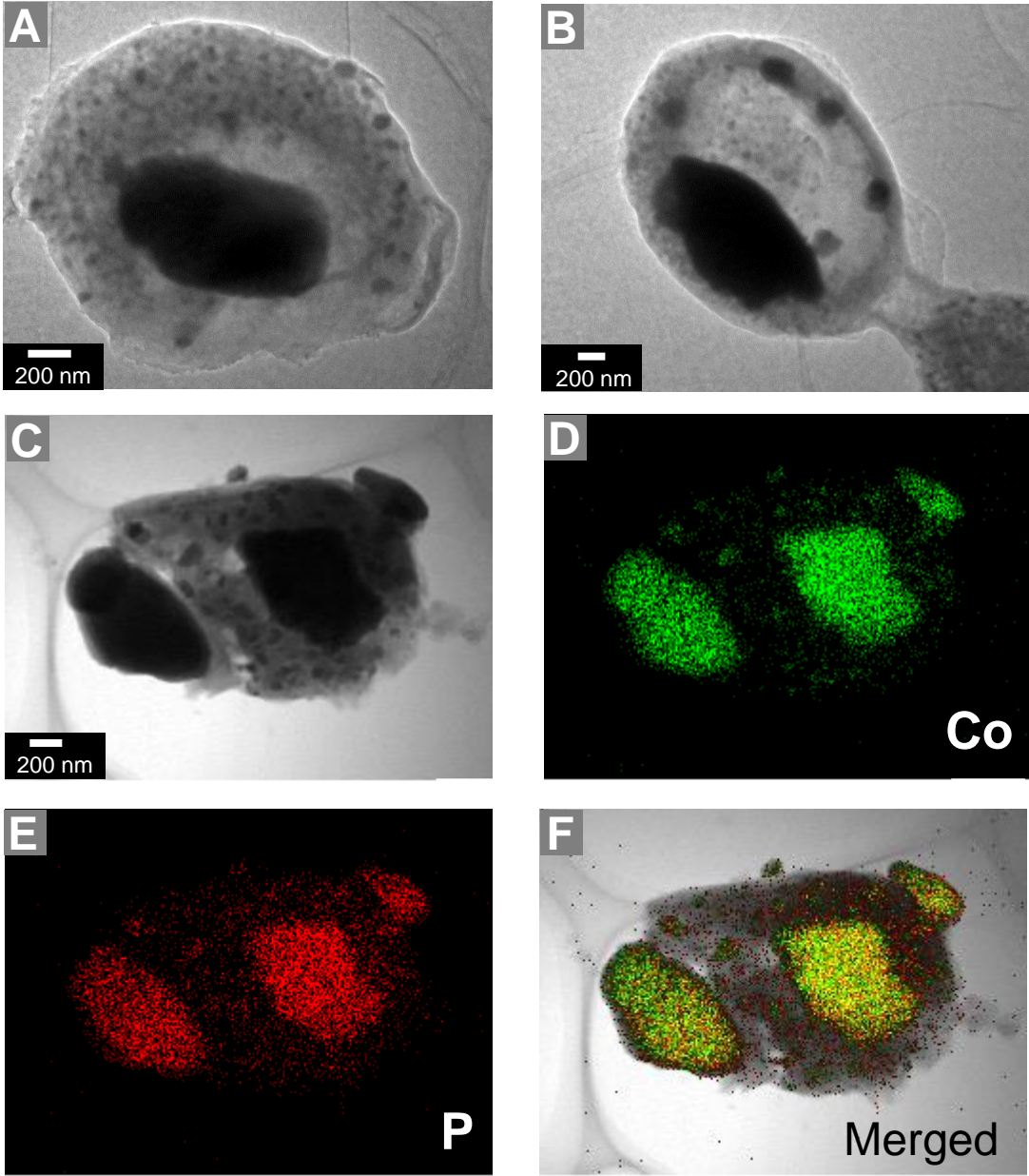

Supplement: Supplementary file 4 — Fig. S4 TEM images (A, B, C) and two-dimensional localization of cobalt and phosphorus on the prepared catalyst (CoP@P-yeast) analyzed using TEM-EDS (D, E, F). [file mmc4.pdf]
