# Supplementary material for: Cobalt phosphide-loaded biochar synthesis using phosphate-accumulating yeast and its application as an electrocatalyst
Source: Biotechnol Rep (Amst). 2025 Jan 9;45:e00874. doi: 10.1016/j.btre.2025.e00874 (PMC11787416; doi:10.1016/j.btre.2025.e00874)
Supplement: Supplementary file 5 [file mmc5.docx]

| Table S1 Specific surface area and conductivity of prepared catalysts | | |
| --- | --- | --- |
| Sample | Specific surface area [m² g⁻¹] | Conductivity [S cm⁻¹] |
| CoP@P-yeast | 278 | 0.085 |
| Co_2_P@yeast | 83 | 0.046 |
| Co_2_P/CoP@yeast(P:0.1 M) | 18 | 0.129 |
| Co_2_P/CoP@yeast(P:0.6 M) | 62 | 0.244 |
| Co_2_P/CoP@yeast(P:1.6 M) | 288 | 0.140 |
| CoP@NA | 249 | 0.003 |
| Pt-C | 125 | 0.174 |
